# Retinotopy drives the variation in scene responses across visual field map divisions of the occipital place area

**DOI:** 10.1167/jov.24.8.10

**Published:** 2024-08-21

**Authors:** Catriona L. Scrivener, Elisa Zamboni, Antony B. Morland, Edward H. Silson

**Affiliations:** 1 Department of Psychology, University of Edinburgh, Edinburgh, UK; 2 Department of Psychology, University of York, York, UK; 3 School of Psychology, University of Nottingham, University Park, Nottingham, UK; 4 York Biomedical Research Institute, University of York, York, UK; 5 York Neuroimaging Centre, Department of Psychology, University of York, York, UK

**Keywords:** retinotopy, scene selectivity, population receptive fields

## Abstract

The occipital place area (OPA) is a scene-selective region on the lateral surface of human occipitotemporal cortex that spatially overlaps multiple visual field maps, as well as portions of cortex that are not currently defined as retinotopic. Here we combined population receptive field modeling and responses to scenes in a representational similarity analysis (RSA) framework to test the prediction that the OPA's visual field map divisions contribute uniquely to the overall pattern of scene selectivity within the OPA. Consistent with this prediction, the patterns of response to a set of complex scenes were heterogeneous between maps. To explain this heterogeneity, we tested the explanatory power of seven candidate models using RSA. These models spanned different scene dimensions (Content, Expanse, Distance), low- and high-level visual features, and navigational affordances. None of the tested models could account for the variation in scene response observed between the OPA's visual field maps. However, the heterogeneity in scene response was correlated with the differences in retinotopic profiles across maps. These data highlight the need to carefully examine the relationship between regions defined as category-selective and the underlying retinotopy, and they suggest that, in the case of the OPA, it may not be appropriate to conceptualize it as a single scene-selective region.

## Introduction

Human scene processing is associated with a trio of brain areas spanning the lateral (occipital place area [OPA]; Dilks, Julian, Paunov, & Kanwisher, 2013; Epstein & Baker, 2019), ventral (parahippocampal place area [PPA]; Epstein & Kanwisher, 1998), and medial (retrosplenial complex or medial place area; Epstein, 2008; Silson, Steel, & Baker, 2016) cortical surfaces, respectively. Considering the OPA specifically, prior work has begun to characterize the visual features that drive its responses. This work highlights the OPA's sensitivity to low-level visual features of scenes, such as the overall distribution of orientations, spatial frequencies, and degree of rectilinearity (Nasr et al., 2011, Nasr, Echavarria, & Tootell, 2014; Watson, Hymers, Hartley, & Andrews, 2016). More recently, voxel-wise responses in the OPA were shown to be predictable on the basis of surface distances and orientations within scenes (Lescroart & Gallant, 2019). Along with clear sensitivity to low-level visual features, the OPA also represents high-level visual features of scenes. For example, the OPA's scene selectivity remains even when rectilinearity is matched between stimuli (Bryan, Julian, & Epstein, 2016). Moreover, advanced multivariate pattern analysis techniques indicate the OPA's sensitivity to high-level properties of scenes, such as landmark identity (Marchette, Vass, Ryan, & Epstein, 2015), scene category (Walther, Caddigan, Fei-Fei, & Beck, 2009), scene shape (Kravitz, Peng, & Baker, 2011), navigational affordances (Bonner & Epstein, 2017), and the mnemonic recall of scenes (Bainbridge, Hall, & Baker, 2021; Baldassano, Beck, & Fei-Fei, 2013; Steel, Billings, Silson, & Robertson, 2021; Steel, Silson, Garcia, & Robertson, 2024).

Previous functional magnetic resonance imaging (fMRI) research also indicates that the OPA overlaps at least five separate visual field maps to varying degrees (Silson, Groen, Kravitz, & Baker, 2016); visual field maps represent visual information retinotopically and are found throughout the human visual system. However, most research regarding the OPA is based on either the average response or the pattern of responses across voxels (or surface nodes) within OPA as a whole. As such, interpretations about the role of the OPA in scene processing have been drawn at the largest spatial scale (i.e., at the level of the OPA itself) rather than the finer-grained level of the visual field maps that subdivide it. Given such diverse recruitment of the OPA, through sensitivity to low- and high-level scene features, a deeper and more comprehensive characterization of the nature of scene processing within the OPA requires investigating the unique contributions of the OPA's visual field maps (Groen, Silson, & Baker, 2017).

**Figure 1.**
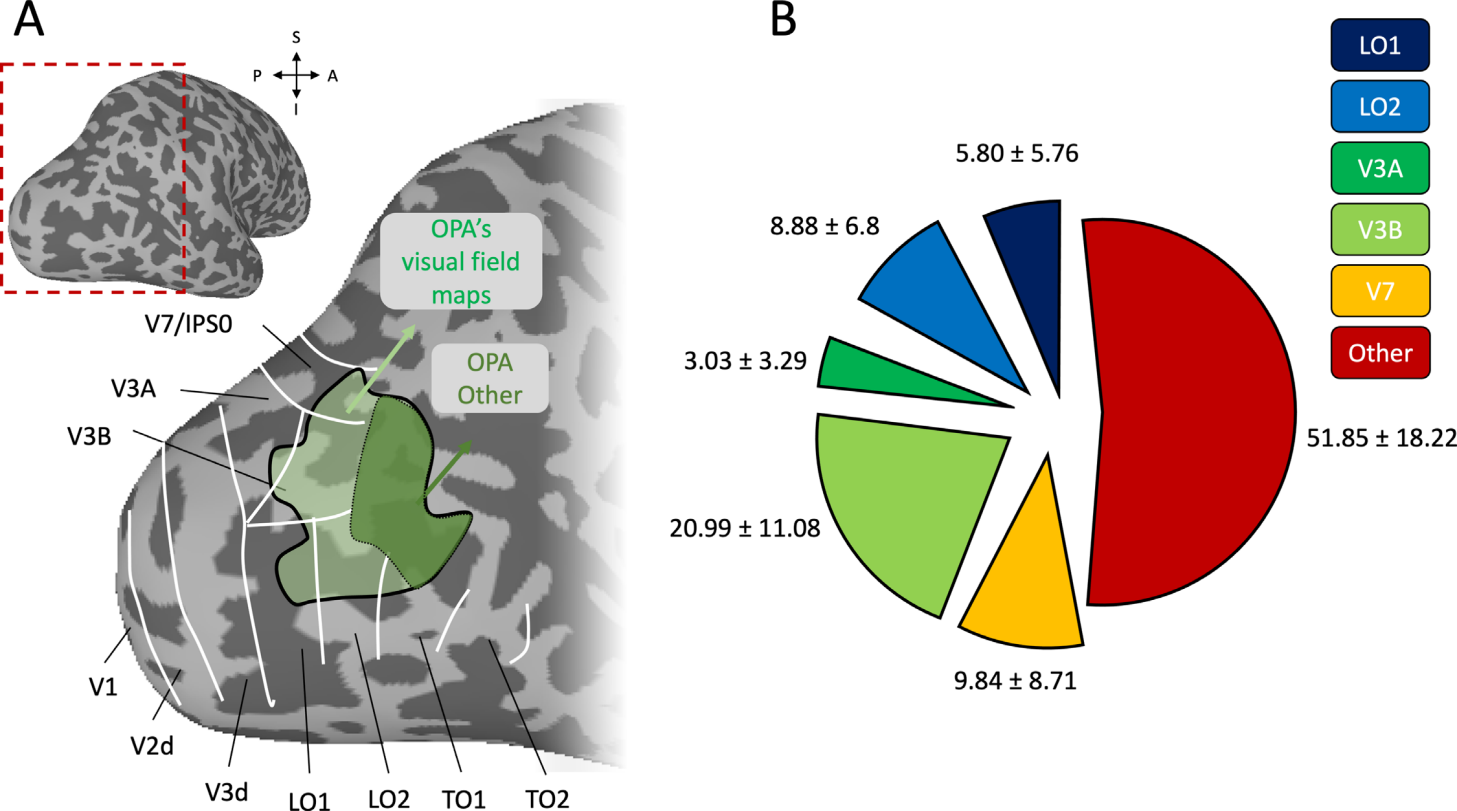
Location of the OPA relative to visual field maps and quantification of the spatial overlap between OPA and visual field map divisions. (A) A lateral view of a partially inflated surface reconstruction of the gray matter from a representative participant. The red-dashed box highlights the lateral occipital portion as in the enlarged inset. The borders of known visual field maps are outlined in white. The group average OPA is overlaid in green. Light green denotes the portion of OPA that spatially overlaps visual field maps, with dark green denoting “OPA Other,” which does not overlap any known visual field maps. (B) The group average (plus/minus standard deviation) percentages of spatial overlap between OPA and the visual field map divisions are shown as sections of a pie chart. Note that these percentages were averaged across hemispheres and participants for display. Consistent with prior work (Silson, Groen, et al., 2016), the OPA had the largest overlap with visual field maps V3B (∼21%), LO2 (∼9%), and V7 (∼10%) but also had a large overlap (∼52%) with a cortical expanse outside of any known retinotopic maps (OPA Other).

The aim of the current study was to test the specific role played by the OPA's visual field maps during scene processing. Here, we measured the responses of the OPA's visual field maps to 96 complex scenes. We predicted that the different maps would represent scenes differently, contributing uniquely to the OPA's overall response profile. Consistent with previous reports (Silson, Groen, et al., 2016), the OPA overlapped visual field maps (LO1, LO2, V3A, V3B, and V7/IPS0) to varying degrees but also spanned a swath of cortex that did not spatially overlap any (as yet) known visual field map—a region we term “OPA Other” (Figure 1).

As anticipated, different patterns of responses were observed across visual field maps. We used representational similarity analysis (RSA) to characterize the representational structure within these visual field maps, by comparing the patterns of response in each map against multiple candidate models spanning different dimensions of scenes, low- and high-level visual features, and navigational affordances. Finally, we considered whether the representational structure was best captured by considering the underlying retinotopic profiles of the visual field maps themselves. Overall, we found no consistent or clear relationship between the pattern of responses measured from each visual field map and the candidate models. Instead, the similarity in response between maps was captured by the similarity of their retinotopic profiles. These data highlight the diversity of responses across the OPA and question whether considering the OPA as a homogeneous scene-selective region remains appropriate.

## Methods

### Participants and testing

Twenty-four participants (mean age: 23.5), seven males, completed an fMRI experiment. Participants included students from the University of Edinburgh and individuals from the surrounding areas. Participants had normal or corrected-to-normal vision and were free from neurological or psychiatric conditions. Written consent was obtained from all participants in accordance with the Declaration of Helsinki and a consent form approved by the School of Philosophy, Psychology and Language Sciences ethics committee of the University of Edinburgh.

### MRI/fMRI imaging parameters

MRI scans were acquired using a Siemens 3T Prisma scanner and 32-channel head coil at the University of Edinburgh Imaging Facility RIE, Edinburgh Royal Infirmary. Participants attended one MRI testing day split into two 1-hour sessions with a break in between (ranging from 15–60 minutes, depending on scanner timetabling constraints). In Session 1, we acquired T1 and T2 weighted structural scans followed by six fMRI runs of population receptive field mapping, lasting 6 minutes each. In Session 2, we acquired a second T1 weighted structural scan to facilitate across-session alignment if needed. This was followed by two runs of a scene localizer task, lasting 5 minutes each, and two runs of an event-related scene perception task, lasting 8 minutes each.

In Session 1, we acquired two structural images: T1 weighted (repetition time (TR) = 2.5 s, echo time (TE) = 4.37 ms, flip angle = 7°, field of view (FOV) = 256 × 256 × 192 mm, resolution = 1 mm isotropic, acceleration factor = 3) and T2 weighted (TR = 3.2 s, TE = 408 ms, flip angle = 9°, FOV = 256 × 240 × 192 mm, resolution = 0.9 mm isotropic, acceleration factor = 2). Functional scans were acquired using a multiecho multiband echo planar imaging sequence (TR = 2; TE = 14.6 ms, 32.84 ms, 51.08 ms; multi-band (MB) factor = 2; acceleration factor = 2; 52/48 interleaved slices; phase encoding anterior to posterior; transversal orientation; slice thickness = 2.7 mm; voxel size = 2.7 × 2.7 mm; distance factor = 0%; flip angle = 70°). To accommodate larger heads, we reduced the number of slices from 52 to 48, which provided greater coverage in the anterior-posterior direction. This change was made from Participant 6 onward. As our analysis is in surface node space and whole-brain coverage was achieved for Participants 1 to 5, who had smaller head sizes, we do not expect this to influence our results.

### Stimuli and tasks

#### Population receptive field modeling

During population receptive field (pRF) mapping sessions, a bar aperture traversed gradually through the visual field while revealing randomly selected scene fragments from 90 possible scenes. During each 36-second sweep, the aperture took 18 evenly spaced steps every 2 seconds (1 TR) to traverse the entire screen. Across the 18 aperture positions, all 90 possible scene images were displayed once. A total of eight sweeps were made during each run (four orientations, two directions). Specifically, the bar aperture progressed in the following order for all runs: left to right, bottom right to top left, top to bottom, bottom left to top right, right to left, top left to bottom right, bottom to top, and top right to bottom left. The bar stimuli covered a circular aperture (diameter = 12° of visual angle). Participants performed a color detection task at fixation, indicating via button press when the white fixation dot changed to red. Color fixation changes occurred semi-randomly, with approximately two color changes per sweep (Silson, Chan, Reynolds, Kravitz, & Baker, 2015).

#### Scene-selective localizer

During each run, color images of scenes and faces were presented at fixation (10 × 10 degrees of visual angle (dva)) in 16-second blocks (20 images per block [300 ms per image, 500 ms blank]). Participants responded via an MRI-compatible button box whenever the same image appeared sequentially (twice per run).

#### Scene perception

During two event-related scene perception scans, participants were presented with 96 complex scenes (12 × 9 degrees of visual angle, 500 ms each) in a randomized order, as in Kravitz et al. (2011). These scenes can be categorized based on their apparent Distance from the viewer (near/far), Expanse (open/closed), and Content (natural/humanmade). Interstimulus intervals (3–7 seconds) were chosen to optimize the ability of the later deconvolution to extract the responses to each scene. Participants fixated centrally and performed an orthogonal fixation color detection task, pressing a button (via MRI-compatible button box) every time the green fixation cross turned red (nine times per scan, set randomly).

### MRI analysis

#### Preprocessing

MRI scans were processed using AFNI (Cox, 1996), Freesurfer, and SUMA (Saad & Reynolds, 2012). Dummy scans were removed from the start of each run (3dTcat). Slice time correction was then performed (3dTshift), aligning each slice with a time offset of zero. The skull was removed from the first Echo 1 scan (TE = 14.6 ms) and used to create a brain mask (3dSkullStrip and 3dAutomask). The first Echo 2 scan (TE = 32.84 ms) was used as a base for motion correction and registration with the T1 structural scan (3dbucket). Motion parameters were estimated for the Echo 2 scans (3dVolreg) and applied to the other echoes (3dAllineate). After completing the standard preprocessing, the data were also processed using tedana (Evans, Kundu, Horovitz, & Bandettini, 2015; Kundu, Inati, Evans, Luh, & Bandettini, 2012) to denoise the multiecho scans (using default options). The tedana optimally combined and denoised output was then scaled so that each voxel had a mean value of 100 (3dTstat and 3dcalc). For the pRF data, an average of the runs was then taken to leave a single time series for further analysis.

The Session 1 structural scans were aligned to the functional data collected in Session 1 (align_epi_anat) and manually checked for accuracy. Functional data collected in Session 2 were aligned to the Session 1 structural data to ensure that all functional data had the same alignment. Freesurfer reconstructions were estimated using both the T1 and T2 scans (recon-all) from Session 1 and the output used to create surfaces readable in SUMA (SUMA_Make_Spec_FS). The SUMA structural was then aligned to the Session 1 experimental structural to ensure alignment with the functional images (SUMA_AlignToExperiment). Surface based analysis was conducted using the SUMA standard cortical surface (std.141).

#### pRF modeling and visual field map delineation

Population receptive fields were estimated using AFNI's nonlinear fitting algorithm (3dNLfim) and the GAM basis function. Full details are provided elsewhere (Silson et al., 2015). The outputs were used to delineate subject-specific visual field maps, which were drawn manually on the SUMA surface using the polar angle and eccentricity parameters (V1, V2d, V2v, V3d, V3v, LO1, LO2, V3A, V3B, and V7/IPS0). These region of interests (ROIs) were then converted into 1D files (ROI2dataset) and the node indices were used to select only those nodes within each visual field map that overlapped with that participant's OPA. Overlapping nodes between neighboring retinotopic maps were removed before analysis such that all included nodes were unique to each visual field map. Almost all visual field maps were identifiable in all participants (see Supplementary Material). A minimum overlap with the OPA of six or more nodes was required for each visual field map to be included in our analyses. Due to the individual nature of the visual field map delineations, OPA definitions, and our inclusion criteria noted above, not all visual field maps overlapped OPA in all participants: left hemisphere (LO1 = 11/24, LO2 = 16/24, V3A = 13/24, V3B = 20/24, V7 = 16/24, OPA Other = 24/24) and right hemisphere (LO1 = 13/24, LO2 = 15/24, V3A = 11/24, V3B = 17/24, V7 = 14/24, OPA Other = 24/24). The number of nodes included in each ROI across participants can be found in the supplementary material as well as a summary of the excluded ROIs and the reason for the exclusion (Supplementary Table S3). Due to the contralateral nature of the visual field representations within these maps, we consider visual field maps within each hemisphere separately throughout.

#### Visual field coverage

The visual field coverage of an ROI includes the locations within a visual field that evoke the greatest response across voxels. We calculated coverage as the best Gaussian population receptive field model for each suprathreshold node within an ROI. We used a max operator that reflects the maximum value from all pRFs within the ROI for each point in the visual field (Winawer, Horiguchi, Sayres, Amano, & Wandell, 2010). The visual field coverage plots represent the group average sensitivity of each ROI to different positions in the visual field. From these data, we quantified two types of visual field biases using the same approach as prior work (Silson, Groen, et al., 2016): contralateral bias (mean contralateral coverage – mean ipsilateral coverage) and elevation bias (mean contralateral upper visual field – mean contralateral lower visual field).

#### Scene-selective regions of interest

To localize the OPA, a general linear model was estimated on the scene face localizer scans using a block design with a 16-second GAM basis function (3dDeconvolve, GAM: 8.6, .547,16). The output of the model was then projected onto the cortical surface (3dVol2Surf) and smoothed with a full-width-half-maximum (FWHM) of 2 mm (SurfSmooth). The OPA ROIs were then drawn manually for each subject on their cortical surface (SUMA draw ROI) after thresholding the contrast of scene versus face stimuli at *t* > 3.5.

#### Scene perception analysis

The activity associated with each stimulus in the scene perception scans was deconvolved using a GAM basis function aligned to the onset of each stimulus. The two runs were modeled together, and each stimulus regressor included two onsets. The data within each participant-specific ROI (OPA from the scene/face localizer, LO1, LO2, V3A, V3B, and V7/IPS0 from the population receptive field mapping) were then extracted for analysis in MATLAB (ConvertDset) using an RSA framework (Nili et al., 2014).

#### Representational dissimilarity analysis

Representational dissimilarity analysis was calculated within each visual ROI on the lateral surface in the left and right hemispheres separately (LO1, LO2, V3A, V3B, and V7), including only the nodes that fell within the larger OPA region. A further ROI was created (OPA Other) using all of the OPA nodes that did not overlap with the visual ROIs. As the ROIs were drawn on a single-subject basis, the number of nodes varied, with some ROIs failing to overlap the OPA in all subjects. For each ROI that met our inclusion criteria, the *t*-values for each stimulus at each node were taken from the event-related general linear model (GLM) output. The pairwise dissimilarities between stimuli *t*-values were then calculated in MATLAB (1 – corr, Pearson, v.2021a MathWorks), organized by their stimulus category, creating a 96 × 96 matrix per ROI. The stimuli were categorized as in Kravitz et al. (2011) based on their Distance (near/far), Expanse (open/closed), and Content (natural/humanmade). To produce group-level representational dissimilarity matrices (RDMs) representing the differences across ROIs, we first computed the pairwise dissimilarity (1 – Spearman's rho) across the 96 × 96 matrices for each ROI in each participant (producing a 5 × 5 RDM), before averaging the dissimilarity matrices across participants. The dissimilarity values were Fisher transformed prior to any secondary analysis (atanh, MATLAB). In the event that a given ROI was not included for a given participant, all correlations involving that ROI were not a number (NaNs) and did not contribute to the final dissimilarity values. For example, if LO1 in a given participant did not meet our inclusion criteria, then all cells in the 5 × 5 RDM involving LO1 would be NaN.

#### Candidate model selection and construction

First, we constructed binary model RDMs to represent the idealized representational structure of responses if they were organized by the dimensions of Distance, Expanse, or Content. This was based on prior work using these same stimuli and models. After these models failed to account fully for the patterns in our data, we decided to consider alternative models. Four additional RDMs were created using models designed to capture low-level visual features (GIST and lateral geniculate nucleus (LGN) models; Groen, Ghebreab, Prins, Lamme, & Scholte, 2013; Oliva & Torralba, 2006), high-level visual features (convolutional neural network [CNN] model), and navigational affordance features (Bonner & Epstein, 2017) of the stimuli. To accurately reflect the chronology of this work, we keep the analyses for the original and alternative models separate throughout.

**GIST model** **.** Image statistics were computed for each image using the gist descriptor (http://people.csail.mit.edu/torralba/code/spatialenvelope/). For each scene, a vector composed of 512 values was created by passing each scene through a series of Gabor filters across four spatial frequencies and eight orientations. The resulting vector characterizes the image in terms of the spatial frequencies and orientations at different spatial locations within the image. We computed the pairwise Euclidean distance between vectors to produce an RDM across stimuli.

**LGN model** **.** Image statistics were computed for each stimulus by taking the output of the model described elsewhere (Groen et al., 2013). In brief, for each image, the model returns two main parameter estimates: spatial coherence (SC), which describes the shape of the contrast distribution, and contrast energy (CE), which describes the scale of the contrast distribution. The RDM was produced by calculating the pairwise Euclidean distance across both of these parameters (SC, CE) for each stimulus.

**CNN model** **.** Stimuli were passed through AlexNet, and the unit activations from the final fully connected layer were extracted prior to computing the pairwise dissimilarity (1 – Pearson's correlation) in unit activations for each scene (Krizhevsky, Sutskever, & Hinton, 2017).

**Navigational affordances** **model.** Following the procedures outlined in prior work (Bonner & Epstein, 2017), we first recorded navigational path trajectories for all 96 scenes from two participants. Participants were instructed to only draw realistic navigable paths from the perspective of the scene. These path trajectories were summed across participants before being passed through 180 one-degree bins to produce an angle histogram per scene. To produce the RDM, we computed the pairwise squared Euclidean distance across the navigability vectors.

**Retinotopic filtering analysis** **.** To quantify the influence of retinotopy on the pattern of response in each ROI, we performed the following analysis. First, we filtered each of the 96 images by the group-averaged visual field coverage plots for each visual field map. Then we computed the GIST descriptors for each of these images and calculating their pairwise distances. This analysis produced a 96 × 96 RDM for each visual field map. Next, we compared the correlation between each ROI scene response RDM and GIST RDMs computed across either (1) the entire image as default and (2) the retinotopically filtered images.

### Statistical analyses

Statistical analyses were conducted using the R Studio package (v1.3) and custom MATLAB code (v.2021a; MathWorks). The dissimilarity values were Fisher transformed prior to secondary analysis (atanh). We used linear mixed models to test for ROI and model interactions (lme4 v27.1; lmerTest v3.1). This was used instead of repeated-measures analyses of variance, which cannot handle missing data points.

## Results

### Quantifying the spatial overlap between the OPA and visual field maps

First, we calculated the proportion of overlap between the OPA and its underlying visual field maps in each participant and hemisphere separately, before averaging across hemispheres. Largely consistent with prior reports (Silson, Groen, et al., 2016), we found that ∼48% of the OPA overlapped five separate visual field maps to varying degrees (Figure 1): V3B (∼21%), V7 (∼10%), and LO2 (∼9%) had the largest overlap, whereas LO1 (∼6%) and V3A (∼3%) had little overlap. The OPA also had a large overlap (∼52%) with no known visual field maps, despite displaying a consistent contralateral visual field coverage (see below).

### Visual field coverage

**Figure 2.**
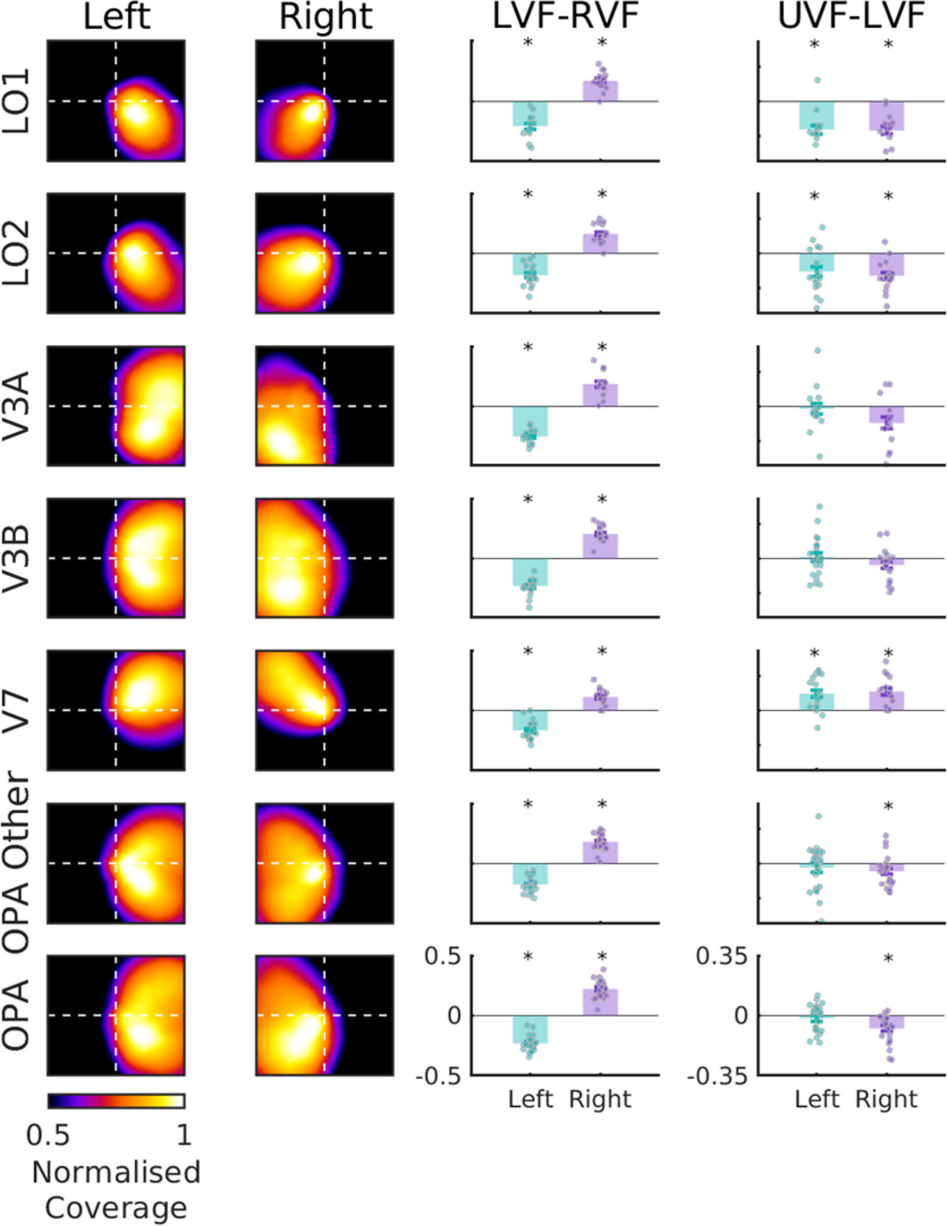
Visual field coverage plots. The heatmaps in the left two panels represent the group average sensitivity of each ROI across positions in the visual field, separately for the left and right hemispheres of the brain. The coverage values were normalized between 0 and 1 to create the coverage plots. The bar plots in the right two panels show the average coverage in each ROI after calculating left minus right visual field (LVF – RVF) and upper minus lower contralateral visual field (UVF – LVF). The values in the bar plots were not normalized. For each ROI, the green bars plot the data in the left hemisphere of the brain, and the purple bars plot the right hemisphere. Each data point represents a single participant. Asterisks indicate *p* < 0.05 in two-tailed one-sample *t*-tests. For the visual field maps (LO1, LO2, V3A, V3B, and V7), we used only the nodes that fell within the larger OPA ROI. For OPA Other, we included the other nodes within the OPA that did not fall into one of the visual field maps. OPA, by definition, includes all of the preceding ROIs.

Next, we computed the visual field coverage for the OPA, OPA Other, and each of the OPA's visual field map divisions using the outputs from the pRF modeling (Figure 2). The visual field coverage plots represent the group average sensitivity of each region to different positions in the visual field. Consistent with prior work (Silson, Groen, et al., 2016; Wandell, Dumoulin, & Brewer, 2007), all ROIs showed a clear contralateral bias. LO1 and LO2 largely represented the lower visual field, whereas V3A and V3B had full hemifield representations. V7 here represented the upper visual field. Both OPA and OPA Other showed a full hemifield representation, albeit biased to the lower visual field (Silson, Groen, et al., 2016). For consistency with prior work (Silson, Groen, et al., 2016), we computed both the contralateral bias (contralateral – ipsilateral) and elevation bias (contralateral upper visual field – contralateral lower visual field) and tested these biases against zero (i.e., no bias) using two-tailed one-way *t*-tests. All visual field maps, as well as OPA and OPA Other, had a significant contralateral bias in both hemispheres (*p* < 0.05). In terms of elevation biases, both LO1 and LO2 were significantly biased toward the lower visual field in both hemispheres, whereas V7 was significantly biased toward the upper visual field. V3A and V3B in both hemispheres did not show a significant bias for either the upper or lower visual field as was anticipated. Despite being numerically larger for the lower visual field, the elevation biases were only significant for OPA and OPA Other in the right hemisphere. This pattern of a stronger lower visual field bias in the right OPA than the left OPA was also found in prior work (see Silson, Groen, et al., 2016; Figure 5). The lower visual field bias in OPA (as a whole) likely reflects its overlap with LO1 and LO2, which showed largely lower quadrant representations in these data.

### Heterogeneity of scene responses across visual field maps

**Figure 3.**
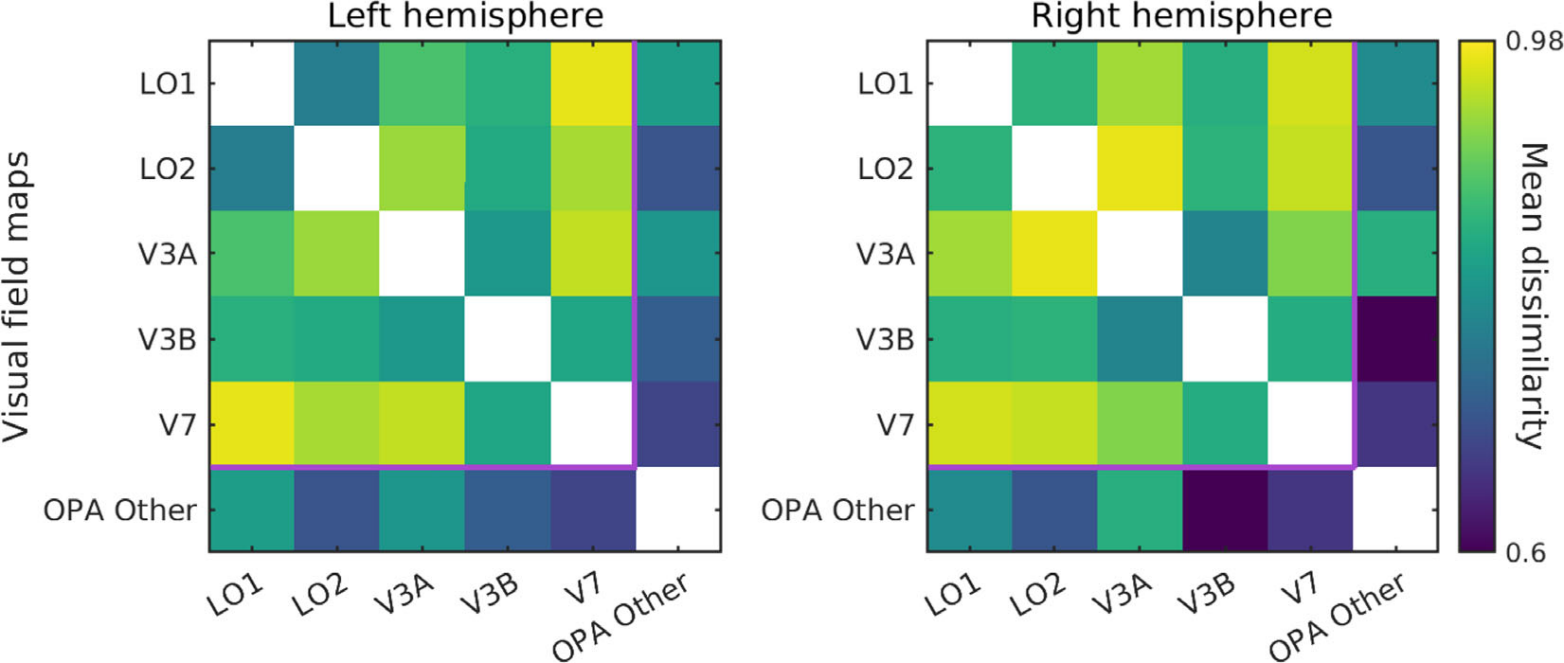
Representational dissimilarity matrices between visual field map divisions of the occipital place area, plus OPA Other. Cells represent the group average pairwise dissimilarity (1 – Pearson's *r*) in the pattern of response between visual field maps in the left and right hemispheres separately. Several groups appear to form; LO1 and LO2 are similar to one another but distinct from V3A, V3B, and V7. V3A is most similar to V3B, whereas V3B is relatively similar to all other ROIs. V7 is distinct from all but V3B. The purple line separates retinotopic subdivisions of OPA from OPA Other.

Next, we examined the heterogeneity in the pattern of responses between these visual field maps, plus OPA Other for completeness. We predicted that the visual field map divisions of the OPA would exhibit different patterns of response. To this end, we first extracted the pattern of responses to each of the 96 stimuli within ROI (visual field maps + OPA Other; Supplementary Figure S1) before comparing these patterns across ROIs (Figure 3). The patterns of responses across the divisions of the OPA were not homogeneous but rather differentiated into what appeared to be three separate clusters: LO1 and LO2, V3A and V3B, and V7. LO1 and LO2 appeared similar to each other but distinct from V3A/V3B and V7, whereas V3A was most similar to V3B only. Patterns of response in V3B were relatively similar to all maps but most similar to V3A, and V7 appeared distinct from all except V3B. Interestingly, the different visual field maps showed a heterogeneous pattern of similarity with OPA Other. Specifically, V3B, V7, and LO2 were the most similar with V3A and LO1 less so. The finding that the patterns of response were not uniform across these ROI divisions of OPA suggests that each cluster (if not each map) may contribute uniquely to the OPA's computations during scene processing.

### Testing scene dimension models

Having established representational differences between the ROI divisions of OPA, we next sought to explain the organizational structure of these representations. Prior work using these same stimuli reported that scene representations in PPA and, to a lesser extent, the OPA were structured by Expanse (i.e., open/closed) (Kravitz et al., 2011). Therefore, we first asked whether the differences between visual field maps could be explained by different responses across the three scene dimensions, Content (humanmade/natural), Expanse (open/closed), and Distance (near/far), using RSA (see Methods). To test the correspondence between the data and the candidate models, we correlated each participant's fMRI RDM with candidate RDMs representing the three scene dimensions above. This was calculated separately in each hemisphere for each visual field map division, as well as in OPA Other. To quantify the relationship between maps, these values were submitted to a linear mixed model (LMM) with ROI (LO1, LO2, V3A, V3B, V7, and OPA Other) and scene dimension (Content, Expanse, and Distance) as factors.

**Figure 4.**
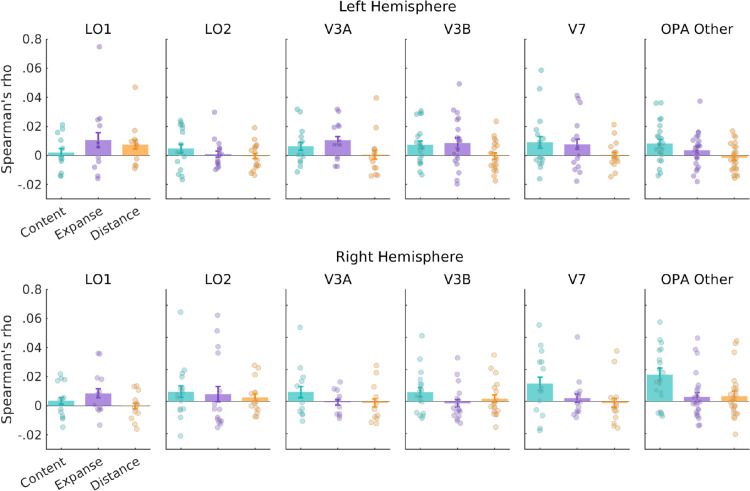
Representational similarity analysis for the OPA's ROI divisions and models for Content, Expanse, and Distance in both hemispheres. Bars represent the average Spearman's rho between the fMRI RDMs for each visual field map (LO1, LO2, V3A, V3B, V7, and OPA Other) and three candidate model RDMs coding for Content (humanmade/natural), Expanse (open/closed), and Distance (near/far) in the left and right hemispheres separately. Error bars represent the standard error of the mean (*SEM*), and each datapoint represents a single subject.

If the heterogeneous pattern of responses reported above could be explained by different responses across these scene dimensions in each ROI, we expected to find a significant ROI by model interaction. However, this interaction was not statistically significant (*F*(10, 264) = 0.85, *p* = 0.57) in the left hemisphere. Instead, we found only a significant main effect of scene dimension, *F*(2, 264) = 5.48, *p* = 0.004 (but not ROI: *F*(5, 276) = 0.95, *p* = 0.44), driven primarily by higher correlations with Expanse on average (Figure 4). Pairwise comparisons (Bonferroni corrected) indicated significant differences for Content versus Distance: *t*(260) = 2.69, *p* = 0.02, and Expanse versus Distance: *t*(260) = 3.01, *p* = 0.008, but not Content versus Expanse: *t*(260) = 0.32, *p* = 1.00. The pattern within each ROI in the left hemisphere was mixed, and only LO1 correlated positively with the Distance RDM model. The close correspondence between LO1 and LO2 in their overall response patterns reported above (see Figure 3) is not reflected here, suggesting that the representational structure within these visual field maps is not organized along either of these scene dimensions. In the right hemisphere, the ROI by model interaction was also not significant, *F*(10, 244) = 0.99, *p* = 0.44. The main effect of scene dimension was significant (*F*(2, 244) = 5.80, *p* = 0.003), driven primarily by higher correlations with Content, on average (Figure 4), but not the main effect of ROI (*F*(5, 259) = 1.02, *p* = 0.40). Pairwise comparisons (Bonferroni corrected) indicated significant differences for Content versus Distance: *t*(244) = 3.27, *p* = 0.003, and Content versus Expanse: *t*(244) = 2.45, *p* = 0.04, but not Expanse versus Distance: *t*(244) = 0.81, *p* = 1.00.

Overall, none of the three scene dimensions could account for the heterogeneous pattern of responses across OPA's divisions (visual field maps + OPA Other). Due to our ROI inclusion criteria (see Methods), different numbers of ROIs contributed to the group average data (e.g., LH LO1 = 11, LH LO2 = 16). We therefore tested whether these missing data could account for the above pattern of results by rerunning our analyses but only including participants with full sets of ROIs in each hemisphere (LH, *n* = 6; RH, *n* = 8). Crucially, the pattern of scene response between ROIs remained heterogeneous even when restricted to complete data sets (see top row Supplementary Figure S2). This pattern of heterogeneity was not captured by our LMM: Importantly, the ROI × Scene dimension interaction remained nonsignificant (*p* > 0.05), with only the main effect of scene dimension reaching significance (*p* < 0.05) in both hemispheres (see Supplementary Table S4 for a full statistical breakdown).

### OPA as a whole

For completeness, we also assessed the extent to which responses in OPA as a whole were organized along the same scene dimensions by submitting the RDM correlations to a linear mixed model with scene dimension as the only factor. This was calculated for each hemisphere separately to mirror the approach taken above. In the left hemisphere, there was a significant main effect of scene dimension (*F*(2, 69) = 6.91, *p* = 0.001), driven by larger correlations with Content and Expanse on average: Pairwise comparisons (Bonferroni corrected) revealed a significant different for Content versus Distance (*t*(46) = 3.67, *p* = 0.001), and Expanse versus Distance approached significance (*t*(46) = 2.32, *p* = 0.07) with no difference for Content versus Expanse (*t*(46) = 1.34, *p* = 0.55). A largely similar pattern was observed in the right hemisphere, with a significant main effect of scene dimension (*F*(2, 69) = 5.82, *p* = 0.004), which was driven by a larger correlation with Content on average: Both Content versus Distance (*t*(46) = 3.13, *p* = 0.009) and Content versus Expanse (*t*(46) = 2.74, *p* = 0.02) were significant (*p* > 0.05, for Expanse versus Distance). In contrast to the ROI subdivisions of OPA, a model coding for both Content (i.e., humanmade/natural) and Expanse (i.e., open/closed) provides potential organizing factors for the responses in left OPA, whereas Content alone appears to account for the responses in right OPA.

Prior work found that responses in the OPA were best explained by a model of Expanse, with no effects for Content when using a bilateral OPA ROI. Interestingly, in our data, the correlation with the Content model remains the largest even when applied to a bilateral OPA ROI (see Discussion and Supplementary Table S5 for details).

### Alternative models

**Figure 5.**
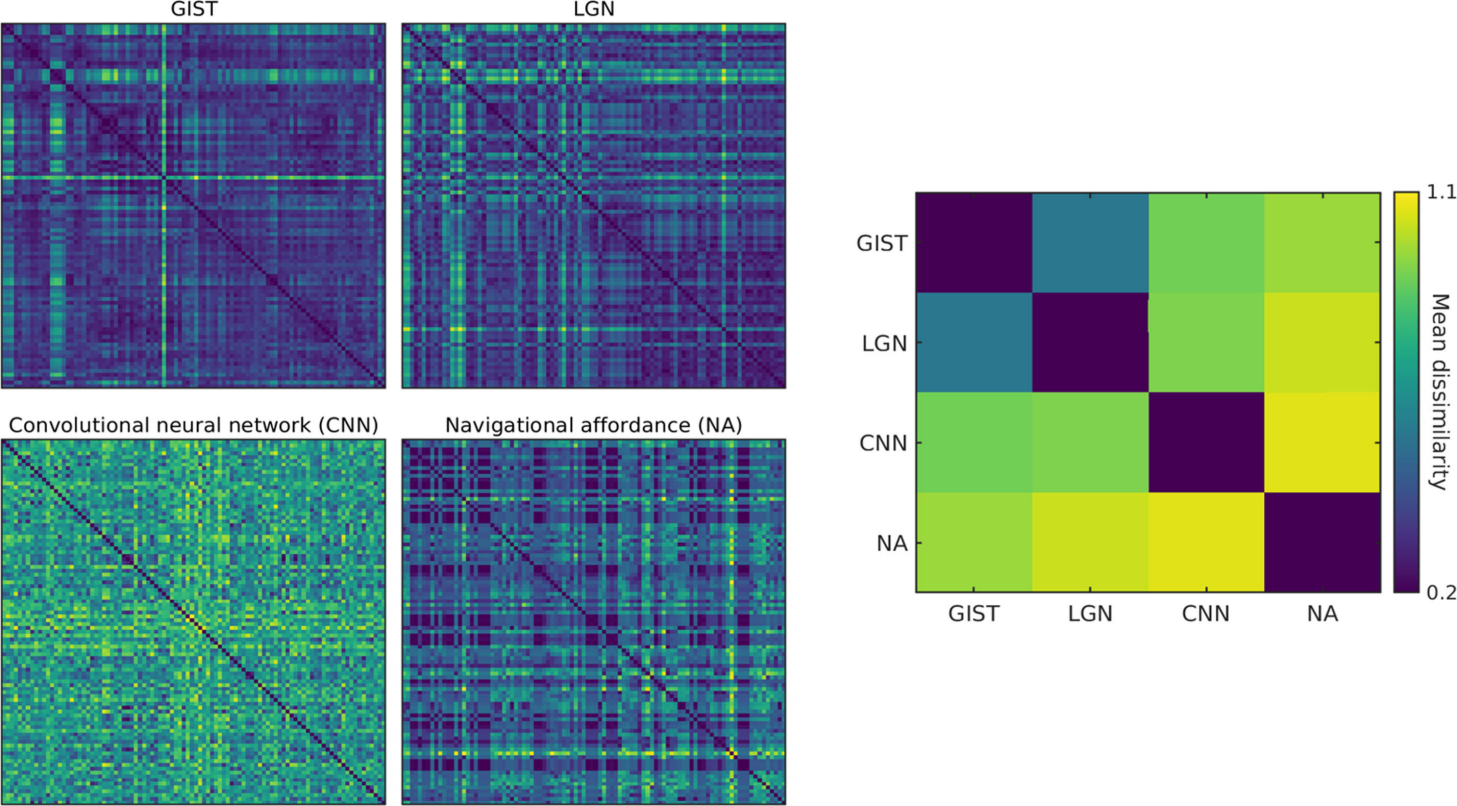
RDMs for four alternative models and the model–model dissimilarities. Left panel: RDM models for the 96 scene images using GIST, LGN, CNN, and NA. Right panel: The pairwise 1 – Spearman's rho between these alternative model RDMs. The low-level image statistic models GIST and LGN were most similar to one another, as expected, but distinct from both the CNN and NA models.

Importantly, the analyses above considered only three candidate models based upon prior work using these same stimuli (Kravitz et al., 2011). It was therefore possible that different candidate models might reveal a consistent representational structure across the ROI divisions of OPA. We explored this possibility by calculating the correspondence between the fMRI RDMs in ROI and four further candidate models (see Methods and Figure 5): two that model low-level image statistics, GIST descriptor (Oliva & Torralba, 2006), LGN statistics (Groen et al., 2013), the final layer of a convolutional neural network (AlexNet; Krizhevsky, Sutskever, & Hinton, 2012), and a model of navigational affordances (Bonner & Epstein, 2017).

**Figure 6.**
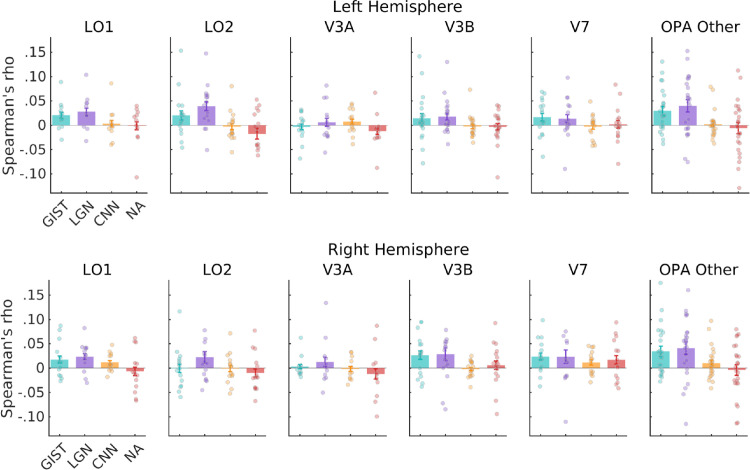
Representational similarity analysis for the OPA's ROI divisions and four alternative models in both hemispheres. Bars represent the average Spearman's rho between the fMRI RDMs for each visual field map and four candidate model RDMs coding for low-level visual properties (GIST and LGN), high-level visual properties (CNN), and NA. Error bars represent the standard error of the mean (*SEM*), and each datapoint represents a single subject.

Consistent with the approach taken above, we first evaluated the correlation between these alternative models and the ROI divisions of OPA in each hemisphere separately before considering OPA as a whole. Similar to our previous analyses, the pattern across the ROI divisions of OPA was varied (Figure 6). To quantify the relationship between maps, these values were submitted to a linear mixed model with ROI (LO1, LO2, V3A, V3B, V7, and OPA Other) and model (GIST, LGN, CNN, and navigational affordances [NA]) as factors. In the left hemisphere, we found only a significant main effect of model (*F*(3, 356) = 12.33, *p* = 1.07-7), driven primarily by higher correlations with the LGN and GIST models on average (ROI by model interaction *F*(15, 356) = 0.91, *p* = 0.54, main effect of ROI *F*(5, 370) = 1.37, *p* = 0.23). Pairwise comparisons (Bonferroni corrected) indicated several significant comparisons: LGN versus CNN, *t*(354) = 4.12, *p* = 0.0003; LGN versus NA, *t*(354) = 5.4, *p* < 0.0001; GIST versus CNN, *t*(354) = 2.77, *p* = 0.03; and GIST versus NA, *t*(354) = 4.06, *p* = 0.0004 (*p* > 0.05 in all other cases). We also found a significant main effect of model in the right hemisphere (*F*(3, 322) = 6.72, *p* = 0.0002), but again no ROI by model interaction (*F*(15, 322) = 0.50, *p* = 0.93) or main effect of ROI (*F*(5, 348) = 1.98, *p* = 0.08). On average, correlations with the LGN model were highest, followed by GIST: LGN versus CNN, *t*(332) = 3.09, *p* = 0.01; LGN versus NA, *t*(332) = 4.05, *p* = 0.0004; GIST versus NA, *t*(332) = 2.88, *p* = 0.02 (*p* > 0.05 in all other cases, Bonferroni corrected). As above, we considered the impact of different numbers of ROIs by rerunning our LMM with only those participants with complete ROIs. Importantly, the ROI × model interaction remained nonsignificant in both hemispheres (*p* > 0.05), with the main effect of model reaching significance in both hemispheres and the main effect of ROI in the right hemisphere (see Supplementary Table S4 for a full statistical breakdown).

### OPA as a whole

Consistent with our approach above, we also considered how these alternative models could account for the patterns of response measured in OPA. In both hemispheres, correlations were highest for the LGN model, followed by the GIST and then CNN models. In contrast, there was a negative correlation with the navigational affordance model in both hemispheres. To quantify these patterns, the fMRI–model correlations for OPA were submitted to a linear mixed model with model as the only factor. In the left hemisphere, the effect of model was significant (*F*(3, 69) = 4.34, *p* = 0.007): Two significant pairwise comparisons were observed (LGN versus CNN, *t*(69) = 2.76, *p* = 0.04; LGN versus NA, *t*(69) = 2.87, *p* = 0.03; *p* > 0.05 in all other cases, Bonferroni corrected). A similar pattern was observed in the right hemisphere with a significant main effect of model (*F*(3, 69) = 3.10, *p* = 0.03). Only the LGN versus NA comparison was significant *t*(69) = 2.72, *p* = 0.04 (*p* > 0.05 in all other cases). These data suggest the responses in OPA are best captured by models that represent low-level visual features.

### Retinotopic similarity between maps accounts for similarity in scene response

At the spatial scale of the OPA's ROI divisions, we failed to observe the anticipated ROI by model interactions, which would have been consistent with the heterogeneous pattern of responses observed between ROIs (Figure 3). Instead, we found only main effects of model, both when considering different scene dimensions and alternative models that capture low- and high-level visual features. How can we reconcile this? One feature of the visual map divisions of OPA, and even OPA Other, is that their visual field representations are not uniform. Indeed, whereas LO1 and LO2 predominantly represent the contralateral lower visual field, V3A and V3B exhibit largely complete hemifield representations (as does OPA Other), with V7, in our data, oversampling the upper visual field (Figure 2).

**Figure 7.**
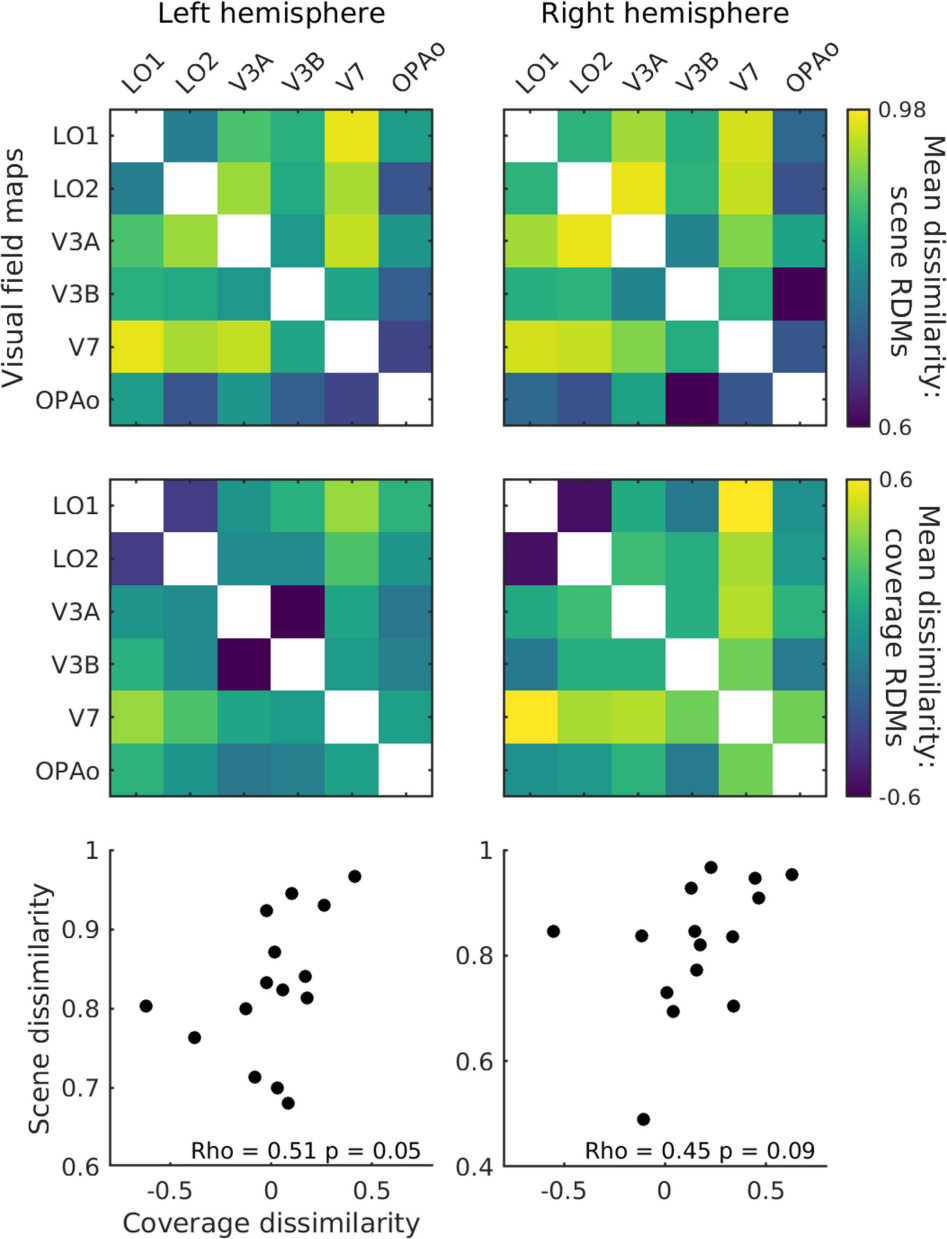
The relationship between scene responses and visual field coverage across ROI divisions of the occipital place area. Top panel: The group average dissimilarity in scene response across visual field maps. Middle panel: The group average dissimilarity in visual field coverage across visual field maps. Bottom panel: The correlation between the lower triangles of the scene dissimilarity and coverage dissimilarity matrices, separately for the left and right hemispheres. OPAo = OPA Other.

Given this, we considered as a final analysis whether the ROI–ROI patterns of response to scenes could be explained simply by the different retinotopic profiles of these ROIs. First, we computed the pairwise dissimilarity in visual field coverage (1 – Pearson's *r* of the visual field coverage plots) between all ROIs in each participant before averaging these correlation coefficients across participants to match the structure of our scene response RDMs (Figure 3). Next, we computed the correlation (Spearman's rho) between these scene response RDMs (Figure 7A) and retinotopy RDMs (Figure 7B) in each hemisphere. Here, the average pattern of similarity in visual field coverage between ROIs was significantly correlated with the pattern of similarity in scene response between those same ROIs in the left hemisphere (LH: rho = 0.51, *p* = 0.05; RH: rho = 0.45, *p* = 0.09; Figure 7C). These data suggest that the structure of responses within the ROI subdivisions of the OPA may be driven by the visual features present at locations in the stimulus represented by those ROIs. That is, the more similar the visual field coverage of two ROI subdivisions of OPA, the more similar the structure of their scene responses and vice versa. Consistent with our approach above, we considered the impact of different numbers of ROIs on this pattern by recomputing the Scene RDM–Coverage RDM correlations with only those participants with complete ROIs. Importantly, the same positive correlations were observed, with significant correlations now in both hemispheres (see Supplementary Figure S2).

**Figure 8.**
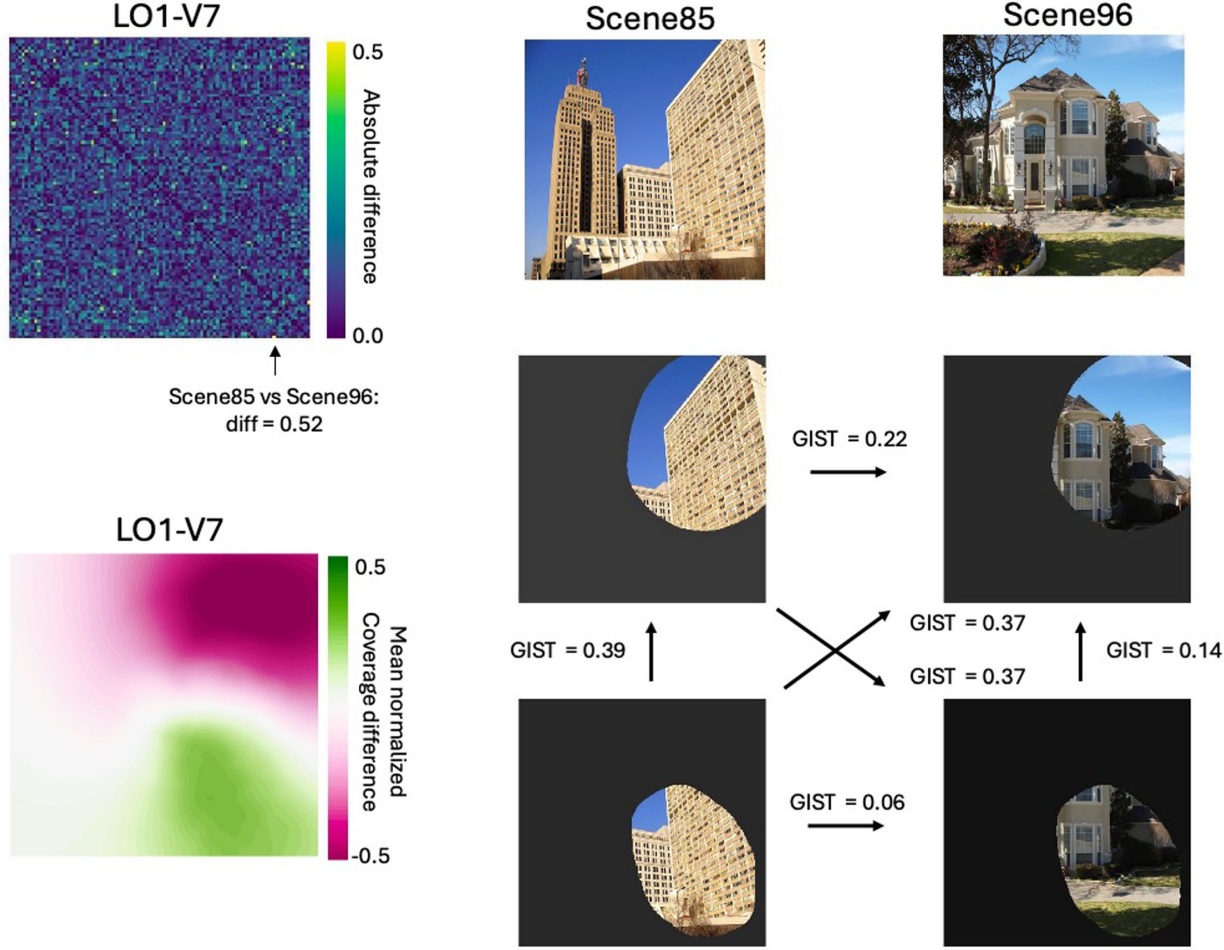
Correlation differences, visual field coverage differences, and retinotopically filtered GIST for LO1 versus V7. Top left: Matrix represents the absolute difference in pairwise correlation between LO1 and V7 in the left hemisphere. The black arrow at cell (x = 96, y = 85) shows the highest absolute difference in dissimilarity between LO1 and V7 (LO1 = 0.04, V7 = 0.92, Difference = 0.52). Bottom left: Matrix represents the mean differential visual field coverage for LO1 versus V7 in the left hemisphere. LO1 exhibits a stronger representation of the lower right quadrant of space (LO1 > V7 = green) with V7 exhibiting a stronger representation of the upper right quadrant of space (V7 > LO1 = pink). Top right: Scenes 85 and 96 from the stimuli presented to all participants. Bottom right: Representations of Scene 85 and Scene 96 after filtering through the group average visual field coverage for V7 (top row) and LO1 (bottom row). Black arrows represent the GIST distance (Euclidean) between pairs of filtered scenes. The smallest GIST distance (i.e., most similar) was for LO1 filtered scenes—almost four times smaller than the V7 GIST distance.

The correlation between similarity in visual field coverage across ROIs and similarity in scene responses indicates a strong influence of retinotopy in driving the response to scenes. To test this interpretation further, we considered whether differences in the visual features at specific retinotopic locations could account for the differences between ROIs. As an indicative example, we focused on LO1 and V7 in the left hemisphere, as these two visual field maps were the most dissimilar on average (Figure 2). First, we calculated the absolute difference between the two group average 96 × 96 RDMs to identify pairs of scenes that differentially drove these visual field maps (see Figure 8, top left panel). This analysis revealed that Scenes 85 and 96 produced the largest absolute difference between LO1 and V7 (dissimilarity difference = 0.52). Visualizing these scenes as a whole suggests that this may be driven by different types of visual information in the upper and lower visual fields, respectively (see Figure 8, top row of right panel). For example, both scenes are dominated by relatively high spatial frequencies at the same relative locations in the lower visual field. In contrast, although Scene 85 has high spatial frequency information throughout the upper right quadrant, that same location is dominated by low spatial frequencies in Scene 96.

Importantly, LO1 and V7 represent very different portions of the visual field. Computing the difference in normalized coverage demonstrates that whereas left hemisphere LO1 represents the lower right quadrant of the visual field, left hemisphere V7 represents the upper right quadrant (see Figure 8, bottom left panel). To quantify whether such differential visual field coverage could account for the difference in correlation between LO1 and V7, we filtered each scene by the group-averaged visual field coverage before computing the GIST descriptors from these filtered images (see Figure 8, right panel). As anticipated, the Euclidean distance in GIST descriptors between LO1 filtered images was almost four times lower than that for V7 filtered images (LO1 D = 0.06, V7 D = 0.22). This indicative example suggests that the different patterns of scene response between ROIs likely reflect the ensemble of visual features at locations represented by that ROI.

To take this one step further, we implemented the retinotopic filtering analysis (see Methods) to all 96 scenes and all ROIs. We filtered each of the 96 images by the group-averaged visual field coverage plots for each ROI before computing the GIST descriptors for each of these images and calculating their pairwise distances. This analysis produced a 96 × 96 RDM for ROI. Next, we compared the correlation between each ROI’s scene response RDM and GIST RDMs computed across either (1) the entire image (as reported in Figure 6) and (2) the retinotopically filtered images.

**Figure 9.**
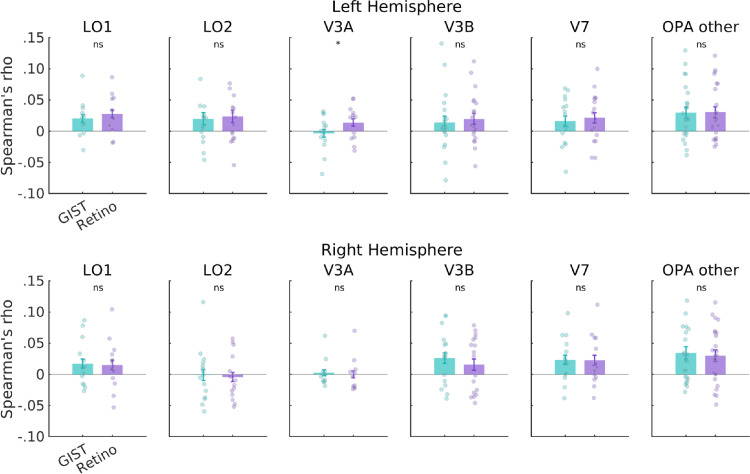
GIST versus retinotopically filtered GIST. Bars represent the mean correlation between each ROI’s scene RDM and two GIST models—one computed across the entire image (GIST: green) and one computed across the image after filtering it by that ROI’s visual field coverage (Retino: purple). In both hemispheres, correlations were similar across both models but were numerically higher for the Retino GIST in the LH. Error bars represent the standard error of the mean (*SEM*) and each datapoint represents a single subject. Asterisks indicate *p* < 0.05 in paired-sample *t*-tests (ns = not significant).

The retinotopically filtered GIST RDMs numerically outperformed the whole GIST RDMs in all ROIs in the left hemisphere and were very similar to the whole GIST RDMs in the right hemisphere (Figure 9). Indeed, a series of paired signed-rank tests revealed only a single significant difference (LH V3A Retino > V3A GIST, *p* = 0.01; *p* > 0.05 in all other cases). It is crucial to note that the Retino GIST RDMs were not significantly worse than the full GIST in any ROI despite being computed using only ∼24% of the total available visual information within the scenes (the most retinotopically relevant 24%).

Taken together, these analyses suggest that the similarity in retinotopy between the OPA's ROI divisions is predictive of the similarity in the pattern of responses to different scenes.

## Discussion

Here we tested the specific role played by the OPA's ROI divisions (visual field maps plus OPA Other) during scene processing using RSA. We compared fMRI responses with models spanning different scene dimensions, low- and high-level visual features, navigational affordances, and retinotopic profiles. As anticipated, we observed a heterogeneous pattern of responses between the OPA's ROI divisions, consistent with the notion that they contribute uniquely to the OPA's overall scene response.

### Retinotopic similarity between visual field maps predicts similarity in patterns of response

Initially, we tested the heterogeneity in the processing of scenes between the ROI divisions of OPA. We computed the pairwise dissimilarity in responses to all scene stimuli in each ROI before correlating those patterns across maps. Consistent with our prediction, the patterns of responses between ROIs were not homogeneous but rather differentiated into what appeared to be three separate clusters within the visual field maps: LO1 and LO2, V3A and V3B, and V7. The clustering of visual field maps and the heterogeneity of responses between maps suggests that each map (or indeed cluster) is likely sensitive to a different feature of scenes. In addition, the different visual field maps showed a heterogeneous pattern of similarity with OPA Other. Specifically, V3B, V7, and LO2 were the most similar, whereas V3A and LO1 were less similar. This pattern of similarity with OPA Other likely reflects that fact that OPA Other has a representation of both the upper visual field (like V7) and the lower visual field (like LO2). The finding that LO1 and V3A were less like OPA Other, despite sharing visual field representations (lower visual field in the case of LO1), might reflect differences in feature tuning.

A critical feature of the OPA's ROI divisions is that their retinotopic profile (as quantified by their visual field coverage) is not uniform. For instance, LO1 and LO2 predominately represent the contralateral lower visual field, whereas V3A and V3B have full hemifield representations, and V7 overrepresents the contralateral upper visual field (in our data, see Figure 7^8^,^9^). In line with previous suggestions (Silson, Groen, et al., 2016), we tested the prediction that the heterogeneous pattern of scene responses between ROIs could be accounted for by the differences in their retinotopic profiles. Indeed, the similarity in scene responses between maps was captured by the similarity in retinotopic profile between maps (at least in the left hemisphere). This was true even when the analysis was restricted to only those participants with complete sets of ROIs (see Supplementary Figure S2). There are (at least) two possible explanations for this pattern: On the one hand, these data could be explained by retinotopy alone. That is, it is possible that these results simply reflect the same visual feature tuning preferences across ROIs but different retinotopic tuning. On the other hand, these data could reflect different visual feature tuning preferences between ROIs that relate to their different retinotopic profiles. We interpret our results as suggesting that ROIs likely undertake quite different visual computations that relate to the types of visual features found typically at those locations within scenes. For example, V3A and V3B contain a representation of the upper visual field not shared by LO1 and LO2 (Figure 2). It is likely, therefore, that V3A and V3B are more sensitive to visual features (both low and high level) that typically occur at those visual field locations. By that logic, the shared lower visual field representation among LO1, LO2, V3A, and V3B may suggest that these maps are similarly sensitive to visual information in the lower visual field. However, there is evidence to suggest that even ROIs with almost identical retinotopic profiles can undertake different visual computations (or use that visual information differently). For instance, LO1 and LO2 are known to be involved in object and shape processing (Larsson & Heeger, 2006; Silson et al., 2013) but show a transition in their dominant representation as measured through multivariate fMRI responses (Vernon, Gouws, Lawrence, Wade, & Morland, 2016). Similarly, V3A and V3B have very similar retinotopic profiles, but whereas V3A is highly sensitive to visual motion (McKeefry, Burton, Vakrou, Barrett, & Morland, 2008; Strong, Silson, Gouws, Morland, & McKeefry, 2017), V3B is not.

We further explored this retinotopic explanation by explicitly taking the retinotopic profiles of the ROIs into account. Specifically, we compared the correlation between the scene RDMs for each ROI and GIST RDMs derived in two ways: (1) using the whole image and (2) when the image was first filtered by the visual field coverage of the ROI. Retinotopically filtering the scene images did not lead to a significant reduction in model performance relative to the entire image, despite removing ∼75% of visual information within the scene. Crucially, the remaining portion of the scene represented the most retinotopically relevant portion for each ROI.

### No tested model can account for the heterogeneity

Having observed the heterogeneity of responses between ROI divisions of OPA, we sought to understand the representational structure of those relationships through RSA. In total, we tested the explanatory power of seven candidate models: three that modeled different scene dimensions (Content, Expanse, and Distance), two that modeled low-level visual features (GIST and LGN), one that modeled high-level visual features (CNN), and a model of navigational affordances (NA). Despite observing numerically different correlations between ROIs, our analyses did not reveal any ROI by model interactions that would have accounted for the heterogeneity of responses between ROIs. Indeed, across the four analyses computed, only a main effect of model was observed. Of course, it is possible that a different set of candidate models may have better explained the heterogeneity in the responses between ROIs (or clusters of ROIs), but this is beyond the scope of the current study and requires further investigation (Groen et al., 2017; Lescroart & Gallant, 2019).

Several important differences between the current data and prior work warrant careful consideration. First, prior work using the same stimuli reported that responses in OPA were best captured by a model of Expanse (i.e., open/closed; Kravitz et al., 2011), although the variance captured by Expanse was weaker in OPA than PPA (Kravitz et al., 2011). Here, when considering OPA as a whole, we find that both Content and Expanse explain similar amounts of variance in left OPA, whereas Content alone explains the variance in right OPA (and in a bilateral OPA ROI). The reasons for this discrepancy are not immediately clear but could reflect differences in the scanning protocol between experiments (EPI vs. multiecho), the number of runs (6 vs. 2), the method of ROI definition (volume based vs. surface based), the criterion for ROI inclusion (nonretinotopic + scene selective vs. scene selective), or a combination of some/all of these factors. We do not interpret our data as more valid than prior work and highlight that the discrepancy offers opportunities for further investigation into the nature of scene processing within OPA (and related regions). Very recently, work employing large-scale generative models to investigate the functional organization of high-level regions of visual cortex including OPA (Luo, Henderson, Wehbe, & Tarr, 2024) found evidence for two clusters within OPA that were more sensitive to indoor and outdoor scenes that contained both humanmade and natural features (i.e., Content), respectively. Given that detailed retinotopic mapping was not conducted, it is difficult to relate this result to the visual field map divisions of OPA or indeed to the location of OPA Other. Nevertheless, these data offer another example of differential responses within divisions of OPA.

Second, prior work has reported a significant correlation between responses in OPA and a model of navigational affordances (Bonner et al., 2017). Here, we do not observe such a correlation either within OPA as a whole (whether considered unilaterally or bilaterally) or any of OPA’s ROI divisions. However, there are important differences between the two stimulus sets that likely explain this discrepancy. For example, the stimuli used by Bonner et al. (2017) were all indoor scenes with clear navigable paths. In contrast, the set of stimuli used in the current study contained both indoor and outdoor scenes that depicted nonrealistic scenarios for navigation (e.g., a bird’s-eye view of a cityscape). Therefore, we do not interpret the lack of correlation with our navigational affordance model as evidence against OPA’s contribution to navigational affordance. Rather, we highlight the possible stimulus dependence of the relationship. Indeed, it is important to recognize that the success of models in capturing brain responses is often contingent on the specific stimuli used.

### The OPA's relationship with visual field maps and OPA Other

Consistent with prior work, we found that almost half (∼48%) of the OPA spatially overlapped at least five visual field maps to differing degrees. The remaining half (∼52%) overlapped more anterior portions of the lateral surface that did not fall within the borders of known visual field maps. Despite this, OPA Other exhibited robust responses during pRF mapping and consistent visual field coverage. This suggests that, although highly sensitive to visual field position, receptive fields within OPA Other are less topographically organized across the cortical surface than those in visual field maps antecedent to it. Further, the lack of evidence for a retinotopic map within OPA Other could be interpreted as an indicator of a weakening of the retinotopic coding scheme in favor of a different coding mechanism. Indeed, our analyses suggest that models based on scene Content (i.e., humanmade/natural) and low-level visual features (GIST, LGN) offer possible coding frameworks for OPA Other. To this point, prior work directly pitting retinotopy and category selectivity against one another reported a posterior-anterior gradient in the strength of these coding schemes (Silson et al., 2021). Posterior portions of the OPA (i.e., its visual field map divisions) more strongly represented retinotopy over category (contralateral bias > category-selectivity bias), whereas more anterior portions outside of OPA's visual field maps (i.e., OPA Other) more strongly represented category over retinotopy (Silson et al., 2021). Notably, the location of this transition between the retinotopic and categorical organizing mechanisms is commensurate with the boundary between the OPA's visual field maps and the location of OPA Other reported here.

The fact that the OPA, as classically defined, spatially overlaps swaths of cortex that both fall within and outside of the borders of known visual field maps means that one should be considerate when defining this area. For example, if one were to take the top 10% of scene-selective voxels within the OPA, they would not necessarily be spatially contiguous and may be spread across multiple maps. In one participant, the voxels with the highest selectivity might fall within LO2 and predominantly represent the lower visual field. In another participant, they may fall within V3B and mostly represent the upper visual field. Across participants, the region defined as the OPA could therefore contain voxels representing different portions of visual space, increasing the between-subject variance, as well as complicating the interpretation of results. This is especially pertinent if the visual stimuli contain markedly different features across the image. Another approach would be to take a set number of voxels centered on the peak voxel of scene selectivity in the OPA, therefore forcing these voxels to be contiguous. However, in our data and prior work (Silson, Groen, et al., 2016), the location of the peak of scene selectivity within the OPA varied across participants. In some, this peak fell within OPA Other; in others, it fell within a particular map (e.g., V3B) or on the border between maps (e.g., LO2/V3B). Thus, even if one selected an ROI based on a set number of contiguous voxels around the peak, the visual field representation of those voxels could still vary quite dramatically between participants.

One way to avoid this issue would be to simply exclude any portion of the OPA that overlaps with known visual field maps (Lescroart & Gallant, 2019; Lescroart, Stansbury, & Gallant, 2015). Such a strategy would avoid the issues raised above about the potential for differing visual field representations among participants. However, this may exclude the location of the most scene-selective portion of the OPA, as the location of the peak moves from participant to participant. Moreover, OPA Other exhibits strong visual field biases, and thus, the extent to which we can remove the influence of spatial biases using this approach is limited. Importantly, we are not advocating one approach over another. Rather, we are recommending that researchers carefully examine the spatial overlap between their category-selective and retinotopic ROIs and take this overlap into account with their analyses and inferences.

## Conclusions

The scene-selective occipital place area spatially overlaps multiple visual field maps, as well as a swath of cortex that does not fall within any given map (OPA Other). The pattern of responses within ROI divisions of the OPA is not uniform. No candidate model we tested could explain the pattern of similarity between these ROIs. Instead, this pattern was correlated with the retinotopic profile of the ROIs. The positive relationship between retinotopic profile and scene responses found here within the OPA's ROI divisions suggests that retinotopy is the driving force behind how similarly two regions represent the same stimulus. These data highlight the importance of considering carefully the relationship between category selectivity and underlying retinotopy and suggest that it is perhaps no longer appropriate to consider OPA as a single homogeneous scene-selective region.

## Supplementary Material

Supplement 1
