# Supplementary material for: Retinotopy drives the variation in scene responses across visual field map divisions of the occipital place area
Source: J Vis. 2024 Aug 21;24(8):10. doi: 10.1167/jov.24.8.10 (PMC11343012; doi:10.1167/jov.24.8.10)

**Supplementary material**

**Table S1: Left hemisphere nodes.** Each row indicates the number of nodes included in each of OPA’s visual field maps for a single participant in the left hemisphere. A minimum overlap with the OPA of six or more nodes was required for each visual field map to be included in our analyses. A value of zero indicates no overlap between the visual field map and the participant’s OPA. Due to the individual nature of the visual field map delineations, OPA definitions, and our inclusion criteria noted above, not all visual field maps overlapped OPA in all participants: lh LO1=11/24, LO2=16/24, V3A=13/24, V3B=20/24, V7=16/24.

**OPA V3a V3b V7 LO1 LO2**

2268 350 114 259 226 286

1727 64 346 60 5 151

1375 91 339 377 90 95

2150 0 307 0 178 301

802 36 524 0 52 43

1062 87 206 210 0 0

1312 30 455 323 0 17

2153 0 0 0 0 0

2603 0 0 0 0 0

1527 65 246 276 267 146

1683 0 115 118 312 415

1813 41 302 81 0 76

1226 0 0 0 0 0

1620 244 617 343 87 113

2715 2 361 0 0 0

2170 0 23 0 0 124

2043 0 69 158 0 30

1005 67 433 75 0 0

599 16 331 214 48 0

684 0 3 114 0 73

2492 14 209 198 222 433

751 0 171 220 0 0

2478 425 571 15 275 773

1625 0 384 0 83 443

**Table S2: Right hemisphere nodes.** Each row indicates the number of nodes included in each of OPA’s visual field maps for a single participant in the right hemisphere. A minimum overlap with OPA of six or more nodes was required for each visual field map to be included in our analyses. A value of zero indicates no overlap between the visual field map and the participant’s OPA. Due to the individual nature of the visual field map delineations, OPA definitions, and our inclusion criteria noted above, not all visual field maps overlapped OPA in all participants: rh LO1=13/24, LO2=15/24, V3A=11/24, V3B=17/24, V7=14/24.

**OPA V3a V3b V7 LO1 LO2**

205 0 0 2 13 26

1582 8 313 14 76 115

1499 60 236 133 17 190

1541 0 891 66 0 149

235 20 0 33 32 17

689 0 39 0 0 0

599 0 7 0 0 36

1858 21 345 12 158 201

2025 23 450 36 507 558

1273 0 0 0 0 0

1263 43 349 201 0 0

778 0 0 0 0 0

738 0 0 0 0 0

731 0 347 0 19 0

945 89 288 5 0 0

1823 6 580 211 60 138

1277 0 211 155 0 96

979 59 190 156 147 100

1577 30 424 371 193 232

698 0 46 291 14 61

1990 0 0 0 0 0

853 0 0 0 0 0

1519 134 746 164 215 38

1439 0 357 32 140 227

**Table S3: ROI exclusion.** Each row indicates the number of visual field maps excluded and the reason for the exclusion. Empty rows indicate that all visual field maps met our inclusion criteria.

**Participant Left Hemisphere Right Hemisphere**

1 2 no overlap

2 1 no overlap

3

4 2 no overlap 2 no overlap

5 1 no overlap 1 no overlap

6 2 no overlap 4 not drawn

7 1 no overlap 3 not drawn

8 5 not drawn

9 5 not drawn

10 5 not drawn

11 1 no overlap 2 not drawn

12 1 no overlap 5 not drawn

13 1 not drawn, 4 no overlap 5 no overlap

14 3 no overlap

15 3 not drawn 2 no overlap

16 1 not drawn, 2 no overlap

17 1 no overlap 1 no overlap

18 2 not drawn

19 1 no overlap

20 2 no overlap 1 no overlap

21 5 not drawn

22 3 no overlap 5 not drawn

23

24 2 no overlap 1 no overlap

**Figure S1: Representational dissimilarity matrices for all ROI divisions of the occipital place area.** Each 96*96 matrix represents the group-average pairwise dissimilarity (1-Pearsons r) in the pattern of response to each of the 96 scenes.


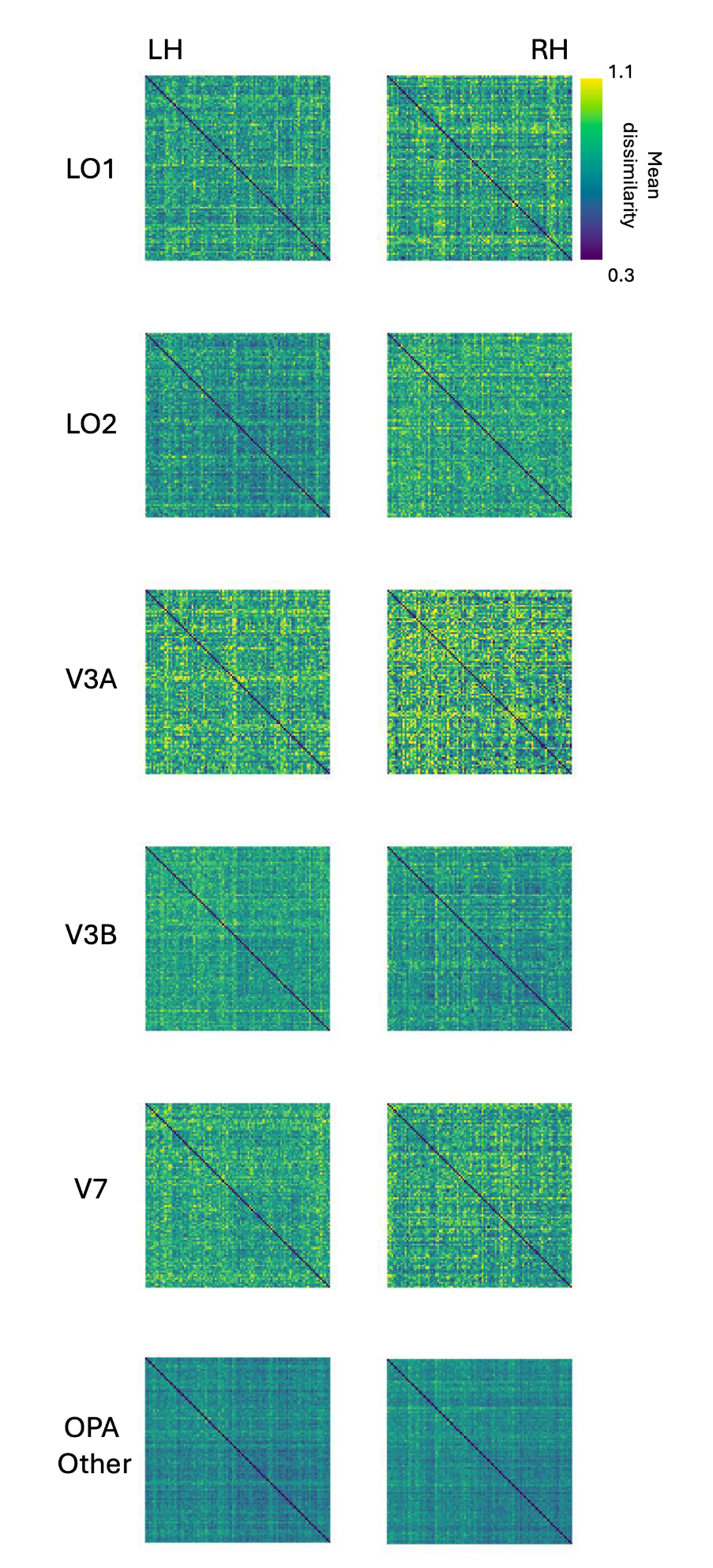


**Figure S2**: **Replication of main figure 7 using only subjects with nodes across all ROIs in each hemisphere (lh = 6, rh = 8)**. Top panel: the group-average dissimilarity in scene response across visual field maps. Middle panel: the group-average dissimilarity in visual field coverage across visual field maps. Bottom panel: the correlation between the lower triangles of the scene dissimilarity and coverage dissimilarity matrices, separately for the left and right hemispheres. OPAo = OPA other.

**
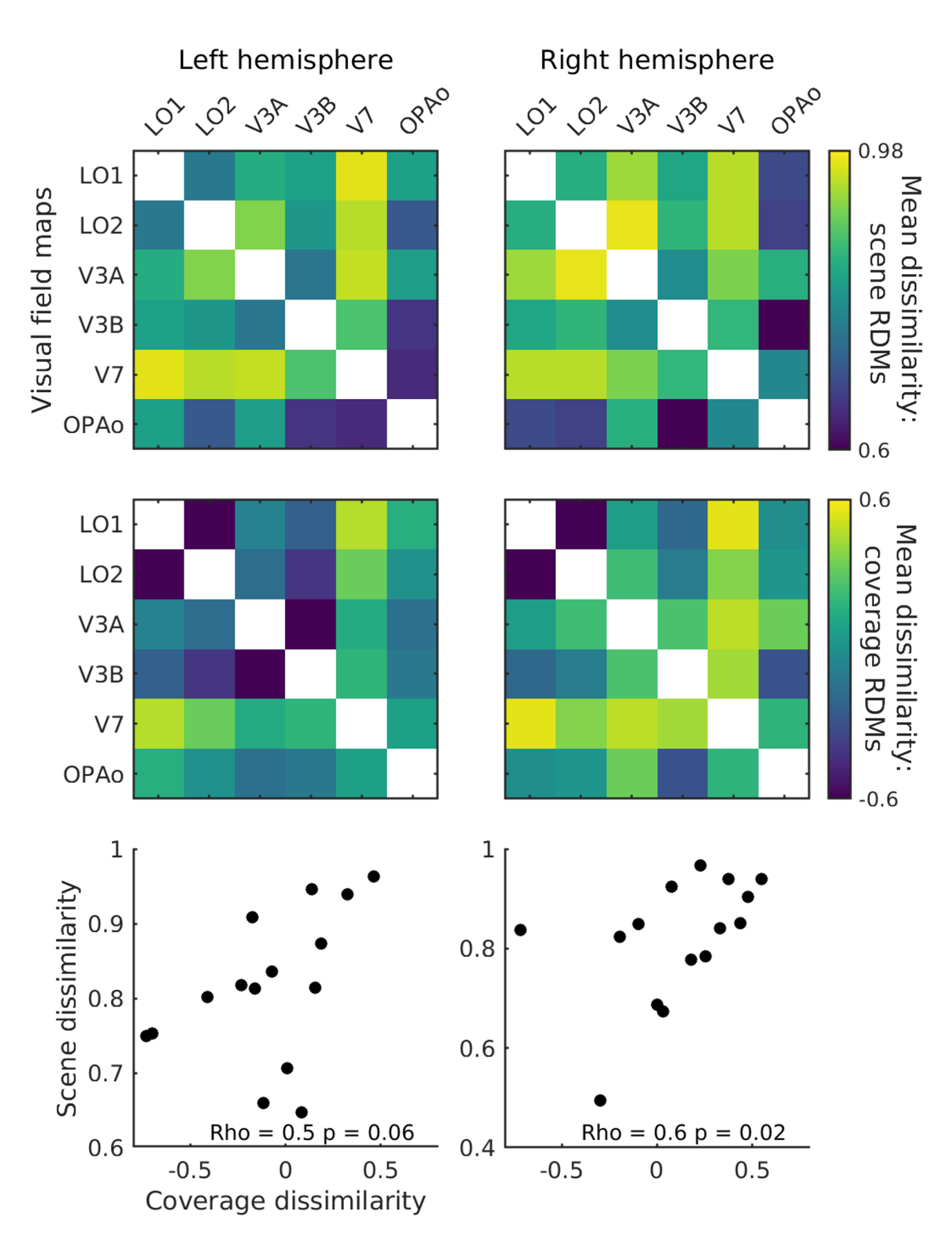
**

**Table S4: LMM results for tests of scene dimensions and alternative models when using only participants with complete datasets:** Table includes degrees of freedom, F-value and p-values results for LMM in both hemispheres.


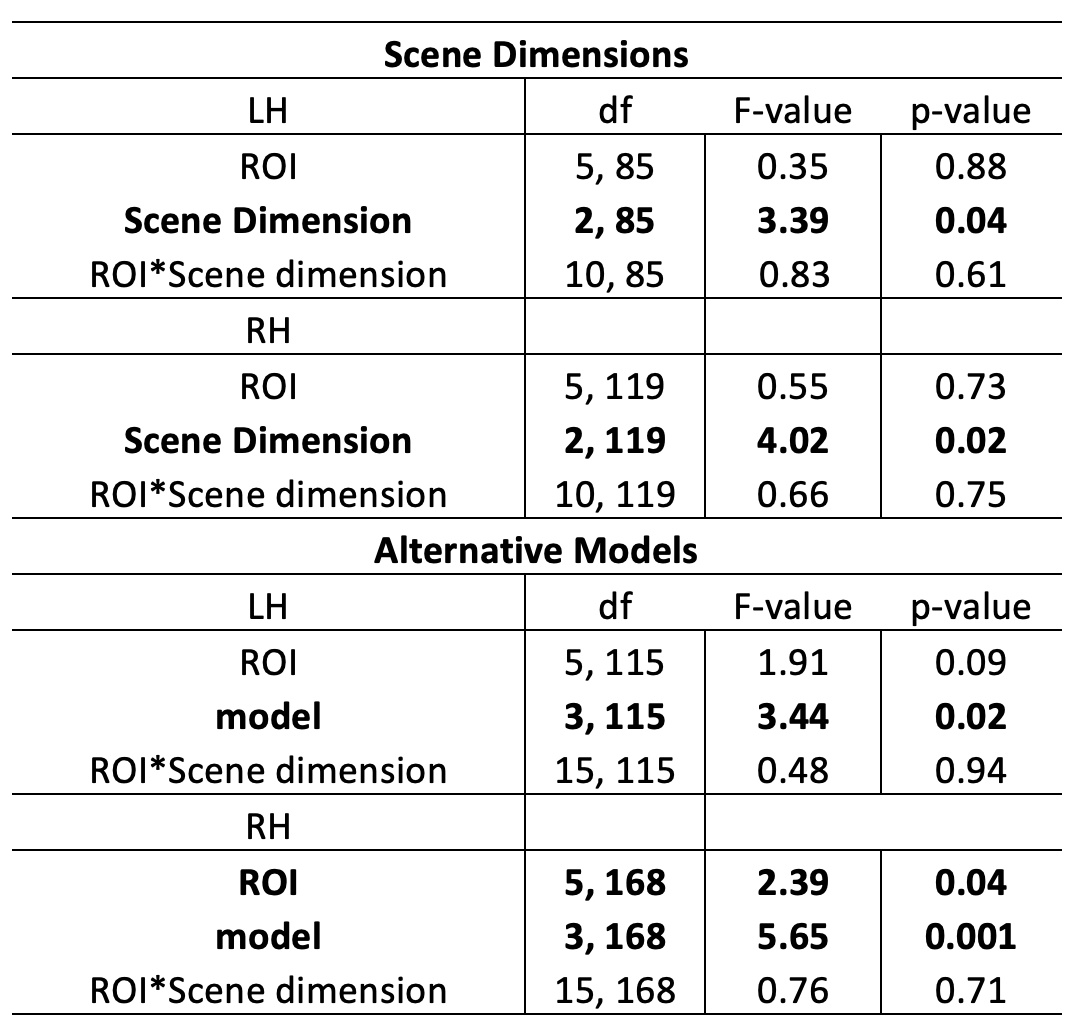


**Table S5: LMM results for tests of scene dimensions and alternative models when using a bilateral OPA ROI:** Table includes degrees of freedom, F-value and p-values results for the main effects and t-values for pairwise comparisons..


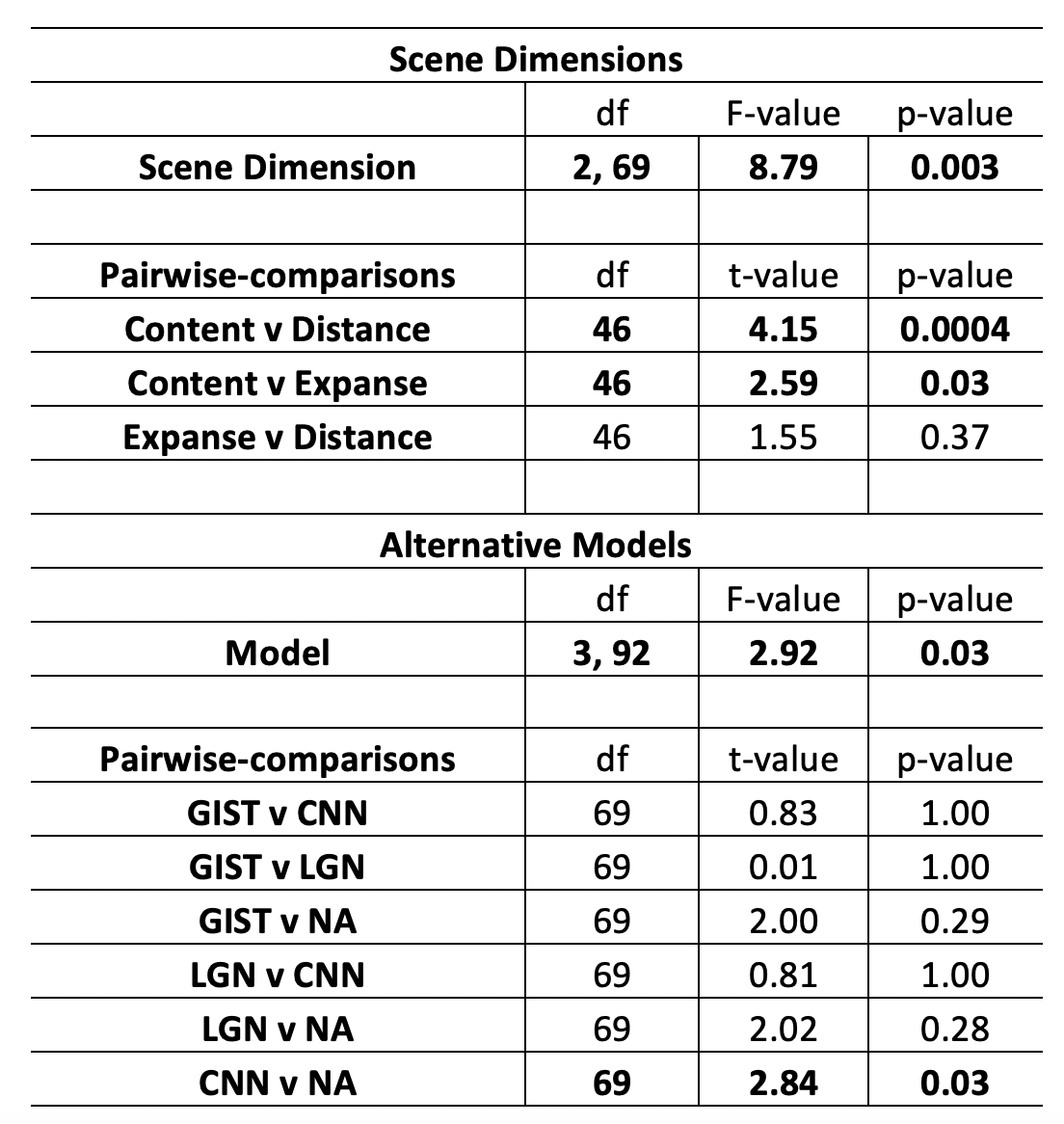

Supplement: Supplement 1 [file jovi-24-8-10_s001.docx]
